# Editorial: Evolution of Reproductive Organs in Land Plants

**DOI:** 10.3389/fpls.2018.00054

**Published:** 2018-01-31

**Authors:** Xin Wang, Zhong-Jian Liu, Borja Cascales-Miñana, José B. Diez

**Affiliations:** ^1^CAS Key Laboratory of Economic Stratigraphy and Palaeogeography, Nanjing Institute of Geology and Paleontology (CAS), Nanjing, China; ^2^Orchid Conservation & Research Center, Shenzhen, China; ^3^Evo-Eco-Paleo, UMR 8198-Centre National de la Recherche Scientifique, University of Lille, Villeneuve d'Ascq, France; ^4^Department of Geosciences, University of Vigo, Vigo, Spain

**Keywords:** reproductive organs, morphology, anatomy, genes, evolution, flowers, hydridization, MADS-box

The evolution of plant life has left its footprints in various ways, either as fossils, as morphology and/or anatomy, or as genes embodied in cells of living organisms. Extant plants are the consequence of over-400-million-years of evolution that are reflected in the innovations of reproductive organs throughout their evolutionary history, such as the occurrence of seeds, ovaries, flowers, inflorescences, and their functions. Thus, understanding different plant reproductive organs and their innovations is essential for the correct interpretation of plant evolution and their phylogenetic relationships. This eBook aims to shed light on the evolution of reproductive organs through papers documenting variations of reproductive organs at different levels, ranging from molecular to morphological, in hopes of triggering further investigations in this field. Below, we highlight the progresses covered in this thematic special volume.

Scientific observation is an active process in which an observer recognizes objects that fall within pre-existing concepts. Considering this framework, it is assumed that (1) perspective decides the outcome of observations and that (2) a new perspective could create the foundation for a new observation. In light of new concepts of sexual reproduction and “Plant Morphogenesis 123,” Bai organized the major morphological traits into five categories, and viewed a plant as a colony of integrated plant developmental units that are each produced via one life cycle. This function-based perspective allows us to view plants in a new way.

Embryogenesis and seed formation are key events in the history of plants. Three papers in this eBook address these issues from different perspectives. To elucidate the seed maturation program, Han et al. surveyed the evolution of the LAFL [L: *LEAFY COTYLEDON1* (*LEC1*) and *LEC1-LIKE* (*L1L*), belonging to *NF-YB* gene family; A: *ABSCISIC ACID INSENSITIVE3* (*ABI3*); F: *FUSCA3* (*FUS3*); L: *LEC2* (*LEAFY COTYLEDON2*). The latter three genes belong to B3-AFL gene family related to embryo development] gene network, which is thought to orchestrate the accumulation of storage compounds and acquisition of desiccation tolerance in seed maturation, among various major plant lineages. Their result indicates that the origin of the embryo-development-related AFL gene family dates back to a common ancestor of the bryophytes and vascular plants. LAFL genes are likely related to spore and seed maturation, and the varying expression patterns of LAFL genes across the major vascular plant lineages may shed new light on the diversifying history of seed plants. Meanwhile, Li et al. and Fang et al. performed related studies using *Adiantum capillus-veneris* from different perspectives. Li et al. characterized the expression patterns of six embryogenesis-associated genes during the somatic embryogenesis of *A. capillus-veneris*. Some of these gene families diversified rapidly in embryophytes, suggesting a rapid evolution of embryogenesis-associated genes in the tracheophyte development. Fang et al. examined the expression pattern of AcLEC1 in *A. capillus-veneris*, and linked sugar treatments with induction of AcLEC1 expression and accumulation of storage products (two characteristic features of seed maturation). Thus, inductive expression of LEC1 homologs during embryogenesis appears to be a key innovation for the origin of seeds.

For long time, the nature and origin of the placenta and carpel (especially in the Centrosperms) has been perplexing, so a rational interpretation is badly needed. Guo et al. conducted a detailed anatomical observation of the vascular bundles within the pistils of *Dianthus chinensis*, and propose a novel interpretation for the nature and origin of these carpels. The vascular bundles are amphicribral in the placenta and collateral in the ovary wall. In light of the discoveries of modern molecular biology, in which development of the placenta and ovary wall have been found to be controlled by separate gene combinations, Guo et al. favors the composite nature of carpels suggested by the Unifying Theory.

The fantastic movement of petals during floral expansion attracts much attention from the general public. Liu et al. studied thermogenic *Magnolia denudata* to explore the biological roles of petal movements. They found that the floral chamber formed by petal movements could facilitate development of the male gametophyte and seed set, and that pollination could accelerate the closure of the inner petals. These findings elucidate the biological roles of petal movements.

Differentiation of flowers within the same inflorescence is an interesting evo-devo phenomenon. Sterile and fertile flowers play important roles in pollinator attraction and sexual reproduction. Lu et al. investigated this regulatory mechanism using *Viburnum macrocephalum* f. *keteleeri*. They developed a *de novo*-assembled floral reference transcriptome, and compared the expression patterns of fertile and sterile flowers at different developmental stages. Combined with morphological and cytological differences between fertile and sterile flowers, they identified many genes and transcription factors potentially involved in regulating the differentiation and development of fertile and sterile flowers within the same inflorescence. This research sheds new light on genes regulating the differentiation of flowers of angiosperms.

Tapetum development is important for the successful reproduction of plants. Previous studies indicated that a “CTCC” sequence within DYT1 (a core regulatory gene of anther development) promoter was indispensable for correct DYT1 expression. Zhou et al. employed site mutation assays to identify the function roles of these nucleotides. They found that the “T” and final “C” of “CTCC” were essential for the temporal and spatial specificity of DYT1 expression. The substitution of two flanking nucleotides of “CTCC” hardly affected the normal promoter function, suggesting that the “CTCC” sequence is a canonical *cis*-element.

Hybridization accompanied by polyploidization and apomixis is a driving force of evolution and speciation in many plants. *Cotoneaster* (Rosaceae), which includes about 150 species, is a good example to study the evolutionary process of hybridization associated with polyploidy and apomixis. Li et al. investigated all the *Cotoneaster* taxa distributed in a small region of Malipo, Yunnan, China. Their study provided convincing evidence for natural hybridization between *Cotoneaster dielsianus* and *Cotoneaster glaucophyllus*. They revealed that all the hybrid individuals were derivatives of one initial F1 via apomixis, and *C. glaucophyllus* served as maternal parent at the initial hybridizing event. Anthropological disturbance appears to have facilitated the hybridization between *C. dielsianus* and *C. glaucophyllus*.

Both compatible and incompatible pollen-stigma interactions are of interest in botany. Zhang et al. applied RNA-seq technology in a comprehensive time-course experiment to explore gene expression during compatible/incompatible pollen-stigma interactions in *Brassica napus*. In contrast to the moderate changes in gene expression both in compatible pollination (PC) and incompatible pollination (PI) within 10 min, drastic changes showed up by 30 min (especially in PI). Stage specific DEGs (Differentially Expressed Genes) were identified. The most highly expressed genes were identified and annotated, and the top 10 highly expressed genes and 37 activated metabolic pathways were revealed. The incompatible response had more complicated signal transduction networks. Niu et al. identified two orchid species displaying gametophytic self-incompatibility (GSI) and investigated their molecular mechanisms by comparative genomics approaches. They analyzed the female determinants of RNase-based GSI in both monocots and eudicots, and identified a novel SI mechanism in orchids.

MADS-box genes are important for floral organ morphogenesis. Two papers in this eBook studied these genes to address the evolution of flowers. Chen et al. analyzed the genomes and large-scale transcriptomes in all the orders of gymnosperms and basal angiosperms. They identified gymnosperm orthologs of 11 genes and characterized a novel subfamily, GMADS, within gymnosperms. ABCE prototype genes have relatively conserved gene number in gymnosperms, but expanded in angiosperms, whereas SVP, SOC1, and GMADS had demonstrated a reversed pattern, namely, expanded in gymnosperms but conserved in angiosperms. The evolutionary history of all MIKC^c^ gene clades apparently reflects the history of seed plants (including gymnosperms and angiosperms). The duplication and expression transition of ABCE model MIKC^c^ genes in the ancestor of angiosperms may have triggered the occurrence of the first flower, providing new insights on the origin of the flowers. Cheng et al. performed a whole-genome survey and identified 34 MADS-box genes in the bamboo species *Phyllostachys edulis*. Their detailed analysis of gene structure and motifs, phylogenetic classification, comparison of gene divergence, and duplication indicated that the ABCDE model is quite conservative throughout monocots and eudicots, and that PheMADS15 might be a regulator of flowering transition in *P. edulis*.

The interaction between the stigma and its insect pollinators is important for successful pollination. Jin et al. studied *Mazus miquelii* (Phrymaceae) with a bilobed stigma to characterize this interaction. They found that larger pollinators transferred more pollen grains to the stigma, causing a rapid stigmatic response and a higher percentage of permanent closures. The permanent closure of a stigma was determined by the size of stigmatic pollen load. The stigma behavior in *M. miquelii* is likely a mechanism of pollinator selection to maximize pollination success.

The Diptera (true flies) are among the most important flower-visiting insects, and they were abundant in the Mesozoic. Zhang and Wang reviewed the fossil record and early evolution of some Mesozoic lower brachyceran flies together with new records in Burmese amber. The fossil records revealed that some flower-visiting groups diversified in the mid-Cretaceous. The evolution of brachyceran groups appears coupled with that of angiosperms.

It is apparent that the limited number of papers in this eBook cannot tell all the stories of evolution of reproductive organs in plants. We are sure future research will enhance our understanding of this topic.

## Author contributions

All authors listed have made a substantial, direct and intellectual contribution to the work, and approved it for publication.

### Conflict of interest statement

The authors declare that the research was conducted in the absence of any commercial or financial relationships that could be construed as a potential conflict of interest.

